# The core genome evolution of *Lactobacillus crispatus* as a driving force for niche competition in the human vaginal tract

**DOI:** 10.1111/1751-7915.14305

**Published:** 2023-07-25

**Authors:** Chiara Tarracchini, Chiara Argentini, Giulia Alessandri, Gabriele Andrea Lugli, Leonardo Mancabelli, Federico Fontana, Rosaria Anzalone, Alice Viappiani, Francesca Turroni, Marco Ventura, Christian Milani

**Affiliations:** ^1^ Laboratory of Probiogenomics, Department of Chemistry, Life Sciences, and Environmental Sustainability University of Parma Parma Italy; ^2^ Department of Medicine and Surgery University of Parma Parma Italy; ^3^ GenProbio Srl Parma Italy; ^4^ Microbiome Research Hub University of Parma Parma Italy

## Abstract

The lower female reproductive tract is notoriously dominated by *Lactobacillus* species, among which *Lactobacillus crispatus* emerges for its protective and health‐promoting activities. Although previous comparative genome analyses highlighted genetic and phenotypic diversity within the *L. crispatus* species, most studies have focused on the presence/absence of accessory genes. Here, we investigated the variation at the single nucleotide level within protein‐encoding genes shared across a human‐derived *L. crispatus* strain selection, which includes 200 currently available human‐derived *L. crispatus* genomes as well as 41 chromosome sequences of such taxon that have been decoded in the framework of this study. Such data clearly pointed out the presence of intra‐species micro‐diversities that could have evolutionary significance contributing to phenotypical diversification by affecting protein domains. Specifically, two single nucleotide variations in the type II pullulanase gene sequence led to specific amino acid substitutions, possibly explaining the substantial differences in the growth performances and competition abilities observed in a multi‐strain bioreactor culture simulating the vaginal environment. Accordingly, *L. crispatus* strains display different growth performances, suggesting that the colonisation and stable persistence in the female reproductive tract between the members of this taxon is highly variable.

## Graphical abstract

Within the lower female reproductive tract, *Lactobacillus crispatus* emerged for its protective and health‐promoting activities. The data presented here clearly pointed out the presence of intra‐species micro‐diversities that could have evolutionary significance contributing to phenotypical diversification by affecting protein domains.

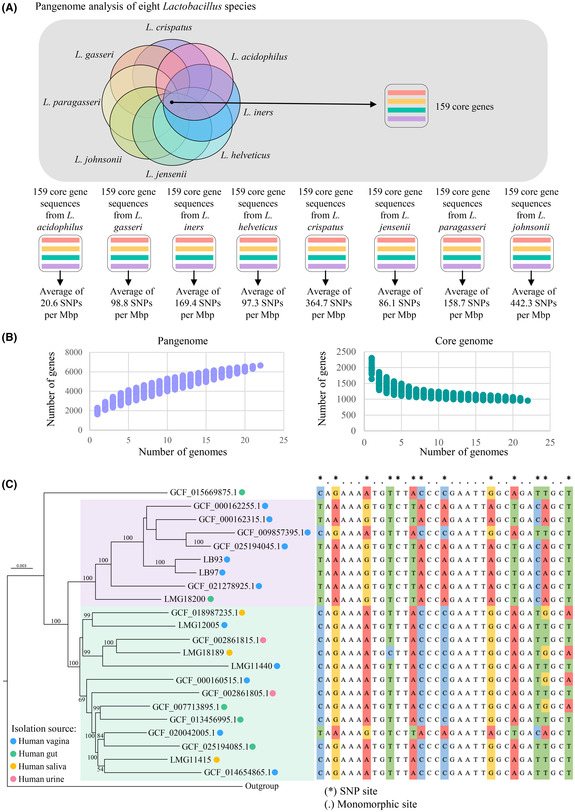

## INTRODUCTION

Bacteria evolved over millions of years to colonise different districts of the human body, for example skin, pulmonary, gastrointestinal and vaginal tracts, giving rise to complex and dynamic populations of microorganisms engaged in close relationships with the human host, referred to as the microbiota (Blum, 2017). In particular, the gut microbiota, with its vastity of microbial genera and species, has attracted increasing interest in the last decades for its ability to impact several aspects of human health, development and systemic physiology from infancy to adulthood (Sommer & Bäckhed, 2013; Strati & Facciotti, 2022; Tarracchini et al., 2022; Turroni, Rizzo et al., 2022; Turroni, van Sinderen et al., 2022; Warner, 2019). In contrast, the vaginal microbiome is typically manifested by a low degree of (bio)diversity and is commonly dominated by members of the *Lactobacillus* genus, such as *Lactobacillus iners*, *L. gasseri*, *L. jensenii* and *L. crispatus*. This latter is regarded as the primary determinant of vaginal health (Lepargneur, 2016; Tachedjian et al., 2017). Indeed, in healthy cervicovaginal microbiota, *L. crispatus* species prevails, producing D‐ and L‐lactic acid, hydrogen peroxide and bacteriocins, which prevent the overgrowth of possible pathogens, hence preventing upper genital tract infections in the host (Sanozky‐Dawes & Barrangou, 2022; Stapleton et al., 2011). For such reason, probiotic supplements based on *L. crispatus* are widely used as vehicles of health‐promoting strains in the vaginal environment (Bohbot et al., 2018; Cohen et al., 2020; Mändar et al., 2023).

Recently, the evolution of the genome sequences of *L. crispatus* species has been studied in relation to its adaptation to the human vaginal niche, underlining strain‐dependent efficiency to grow on glycogen as well as to inhibit pathogens (Abdelmaksoud et al., 2016; Argentini et al., 2022; Mendes‐Soares et al., 2014; Ojala et al., 2014; Puebla‐Barragan et al., 2021; van der Veer et al., 2019). Moreover, besides the human vaginal tract, *L. crispatus* have also been identified and isolated from various (sub)niches, ranging from healthy poultry gut to various districts of the human body, including the oral cavity, rectum, and urinary tract, highlighting within‐species genetic diversity and variegated metabolic capabilities (France et al., 2016; Mancabelli et al., 2021; Pan et al., 2020; Zhang et al., 2020). Taken together, this evidence suggests the presence of distinct evolutive trajectories underlying the observed phenotypic diversification within this species. However, comparative genomic analyses involving chromosome sequences of *L. crispatus* species have often focused on the relationship between the presence/absence of accessory genes and a particular phenotype (Pan et al., 2020; Zhang et al., 2020), overshadowing the importance of mutations to within‐species evolution (Juhas et al., 2011; Martínez‐Carranza et al., 2018; Rousset et al., 2021).

In this framework, the aim of this study is to evaluate genome sequence variations at the single nucleotide level within protein‐encoding genes shared across non‐identical *L. crispatus* chromosomes, providing a close‐up view of genetic (micro)diversity, which can contribute significantly to strain diversification within this species. In addition, to investigate the possible implications of the identified genetic differences in the *L. crispatus* intra‐species competition within the vaginal microbiota, we performed in vitro experiments consisting of carbohydrate growth assays involving *L. crispatus* multi‐strain co‐cultivation in a bioreactor simulating the vaginal tract.

Our findings revealed inter‐strain genotypic variation and phenotypic differences between *L. crispatus* strains, highlighting distinct evolutionary developments that may provide this species with differential abilities to long persist and predominate in the human vaginal tract.

## EXPERIMENTAL PROCEDURES

### Isolation of *L. crispatus* strains and retrieval of publicly available genome sequences

Candidate *Lactobacillus* strains were obtained from an isolation effort performed in a framework a previous study.

Identification of newly isolated *L. crispatus* strains was achieved through the Matrix‐Assisted Laser Desorption/Ionization Time‐of‐Flight Mass Spectrometry (MALDI‐TOF MS) Biotyper Sirius (Bruker) using the manufacturer's software FlexControl and the MALDI‐Biotyper software (MBT). In detail, a single bacterial colony grown on MRS agar was transferred onto a spot of the MSP 96 target polished steel BC MALDI target plate (Bruker). Subsequently, the bacterial sample was overlaid with 1 μL of matrix solution containing 10 mg/mL HCCA (a‐cyano‐4‐hydroxycinnamic acid, Sigma‐Aldrich) resolved in 50% acetonitrile (Carlo Erba) and 2.5% TFA (trifluoro‐acetic acid, Carlo Erba) and air‐dried (Schulthess et al., 2014; Werner et al., 2012). The MALDI target plate was then introduced into the spectrometer for automated measurement and data interpretation. The mass spectra were processed with the MALDI Biotyper 3.0 software package (Bruker) containing reference spectra, including different lactobacilli species. According to the criteria recommended by the manufacturer, a score of ≥2.000 indicates a significant similarity between the obtained spectrum and the database entry. Each sample was analysed in duplicate (two spots for each sample).

The default parameter settings are as follows: positive linear mode, laser frequency 200 Hz, ion source 1 = 19.84 kV, ion source 2 = 18.07 kV, Bruker's MBT_FC and MBT_AutoX methods, mass range: 2000–20,000 Da. Moreover, before analysis, calibration was performed with a bacterial test standard (Bruker) containing an extract of *Escherichia coli* DH5 alpha.

A total of 34 *L. crispatus* strains were identified and taken forward for whole genome sequencing. In addition, seven *L. crispatus* strains isolated from human biological samples were purchased from international bacterial collections. To perform chromosomal DNA extraction, the 41 *L. crispatus* strains were cultivated in MRS broth supplemented with 0.05% (wt/vol) L‐cysteine hydrochloride in an anaerobic atmosphere at 37°C (2.99% [vol/vol] H_2_, 17.01% [vol/vol] CO_2_, and 80% [vol/vol] N_2_) for 12 h. Subsequently, cells from 10 mL of the culture were harvested by centrifugation at 6000 rpm for 8 min, and the obtained cell pellet was used for DNA extraction using the GenElute bacterial genomic DNA kit (Sigma‐Aldrich) following the manufacturer's guide.

Furthermore, 200 *L. crispatus* genome assemblies with completeness >95% derived from strains isolated from biological material of human subjects were retrieved from the NCBI database.

### Genome sequencing, assembly, and annotation

The DNA extracted from the 41 *L. crispatus* strains was subjected to whole‐genome sequencing using MiSeq (Illumina) at GenProbio srl, Parma, Italy (www.genprobio.com) according to the supplier's protocol (Illumina). Individual genome libraries were generated using the Nextera XT preparation kit and loaded into a 600‐cycle (250‐bp paired‐ends) flow cell version 3 (Illumina). Raw DNA sequence reads (fastq files) obtained from genome sequencing were assembled using the MEGAnnotator pipeline (Lugli et al., 2016). Briefly, SPAdes software was used for de novo assembly of the genome sequences with the pipeline option “‐‐carefull” and a list of k‐mer sizes of 21; 33; 55; 77; 99; 127 (Bankevich et al., 2012) and protein‐encoding genes were predicted for contig greater than 1000 bp using Prodigal (Hyatt et al., 2010). Functionally, annotation of the predicted genes was achieved through RAPSearch2 (cut‐off *E* value, 1 × 10^−5^; minimum alignment length, 20 amino acids) (Zhao, Tang et al., 2012) and hidden Markov model (HMM) profile searches (cut‐off E value, 1 × 10^−10^; http://hmmer.org/) performed against the NCBI nr database and the manually curated Pfam‐A database, respectively. Moreover, tRNA genes were determined using tRNAscan‐SE version 1.4 (Lowe & Eddy, 1997), and rRNA loci were identified with RNAmmer version 1.2 (Lagesen et al., 2007).

Furthermore, to obtain comparable quality standards for the analysed genomes, all the 200 *L. crispatus* genomes retrieved from the NCBI database were re‐annotated employing the same approach based on MEGAnnotator pipeline used for the 41 *L. crispatus* genomes decoded in the current study.

### Pangenome analyses and phylogenomic tree reconstruction

All pangenome calculations were performed using PGAP [PanGenomes Analysis Pipeline, (Zhao, Wu et al., 2012)] as described previously (Lugli et al., 2019; Tarracchini et al., 2020). In detail, orthologous protein sequences were identified in genome sequences using BLAST analysis (cut‐off *E*‐value = 1 × 10^−5^; 50% identity over at least 80% of sequence coverage) and then organised into functional Clusters of Orthologous Groups (COGs) through the MCL algorithm (graph‐based Markov clustering algorithm) using the gene family (GF) method. Pangenome profiles were produced through an optimised procedure integrated into the PGAP software, based on a presence/absence matrix including all COGs identified in the given genomes. The concatenated protein sequences of core genes were aligned using Mafft v7.453 (Katoh et al., 2002) and then employed to build correspondent phylogenomic trees through the neighbour‐joining method in ClustalW version 2.1. Visual core genome‐based phylogenomic trees were developed using FigTree software (http://tree.bio.ed.ac.uk/software/figtree/).

### Single‐nucleotide polymorphism identification

The species‐specific level of polymorphisms within the *Lactobacillus* genus was assessed exploiting the identified core gene set shared between *L. crispatus* and seven different *Lactobacillus* species. In detail, 159 core‐shaping genes were concatenated and aligned using the multiple genome aligner Mafft v7.453 (Katoh et al., 2002). Nucleotide variants at each sequence position were then extracted through the SNP‐sites program (version 2.5.1) (Page et al., 2016). Assuming that, unlike sequencing errors, real genetic variants should be observed in a quite number of independent genomes assembly, we considered only sequence positions in which two or more alternatives were observed in at least 20% of genome collection. The number of intra‐species SNPs obtained for each *Lactobacillus* species was converted into SNPs per Mbp to account for variation in genome length.

In a similar fashion, both concatenated and individual gene nucleotide sequences comprised within the *L. crispatus* core genome were aligned with Mafft v7.453 and parsed with the SNP‐site software. For these analyses, the genome sequence of the most divergent *L. crispatus* strain (assembly number GCF_015669875.1) was used as reference sequence. Synonymous and non‐synonymous nucleotide variations were discriminated using the ParaAT 2.0 software (Zhang et al., 2012) combined with KaKs Calculator 3.0 toolkit (Zhang, 2022). Whole‐genome SNPs were extracted by combining the short‐reads aligner BWA and the VarScan tool (version 2.3.6).

### Carbohydrate growth assay

In vitro growth assays with different carbon sources, such as starch, amylopectin, pullulan, maltodextrin, glycogen and mucin, were performed on selected *L. crispatus* strains, that is LB97, LMG11440, LMG18200 and LMG11415. In detail, the four *L. crispatus* strains were cultivated overnight on a semisynthetic MRS medium supplemented with 0.05% (w/vol) L‐cysteine hydrochloride at 37°C under anaerobic conditions. Subsequently, cells were diluted in MRS without glucose to obtain an OD_600 nm_ = 1, and 15 μL of the diluted cells were inoculated in 135 μL of MRS without glucose supplemented with 1% (wt/vol) of a particular sugar in a 96‐well microtiter plate and incubated in an anaerobic cabinet. Specifically, each carbohydrate was dissolved in MRS without glucose previously sterilised by autoclaving at 121°C for 15 min. Subsequently, the obtained solutions were sterilised using a 0.2 μm filter size before use. Cell growth was evaluated by monitoring the OD at 600 nm using a plate reader (Biotek). Each plate was read in discontinuous mode, with absorbance readings performed thrice at 3‐min intervals after 48 h of growth, and each reading was ahead of 30s of shaking at medium speed. Cultures were performed in triplicates for each strain, and the resulting growth data were expressed as the average OD_600nm_ of these independent biological replicates. Carbohydrates tested in this study were purchased from Merck and Fisher Scientific, ACROS Organics and include soluble starch from potato, amylopectin from maize, pullulan, maltodextrin, glycogen from beef liver, mucin from porcine stomach. The semisynthetic MRS medium was used as the control medium.

### Co‐culture using a bioreactor system

The four selected *L. crispatus* strains (reported above) were grown anaerobically at 37°C for 24 h in simulated vaginal fluid (SVF) (Pan et al., 2020) to adapt the microorganisms to the medium. Next, revitalised cells were inoculated in a bioreactor system (Solaris Biotech Solutions) to obtain a final concentration per bacterial strain of 5 × 10^6^ cells/mL in 400 mL of SVF. The co‐culture of the four *L. crispatus* strains was performed with a non‐continuous supply of the growth medium for the first 12 h to stabilise the microbial community. Subsequently, the cultivation was shifted to a continuous mode to provide fresh SVF medium and continued for 48 h under anaerobic conditions at 37°C with a mechanical agitation set at 180 rpm. In addition, the pH was maintained at 4.5 by adding 2.5 M NaOH to mimic the pH of the human vaginal environment (Pan et al., 2020). During bacterial growth, culture aliquots were collected at 10, 24, and 48.

### DNA extraction and shotgun metagenomic sequencing

Each aliquot collected from bioreactor cultivation was subjected to DNA extraction using the ZymoBIOMICS DNA Miniprep Kit (Zymo Research, D4300) following the manufacturer's instructions. Then, after assessing DNA concentration and purity using a BioPhotometer D30 (Eppendorf, Germany), each DNA sample was sequenced by GenProbio srl, Parma, Italy (www.genprobio.com) employing next‐generation sequencing technique (shotgun metagenomic sequencing). The preparation of DNA libraries was performed using the Nextera XT DNA sample preparation kit (Illumina) according to the manufacturer's instructions, using 1 ng of DNA from each metagenomic sample. The isolated DNA underwent fragmentation, adapter ligation and purification. The ready‐to‐go libraries were pooled equimolarly and diluted to a sequencing concentration of 650 pM. On‐board DNA denaturation and sequencing were performed on a NextSeq 2000 instrument (Illumina), according to the manufacturer's instructions, using the 2 × 150 bp NextSeq 1000/2000 P2 Reagents (300 Cycles) v3 and spike‐in of 1% PhiX control library. Whole‐metagenome shotgun (WMGS) sequencing of the three bioreactor culture aliquots produced an average of 21,861,838 ± 7,995,330 paired‐end 150 bp reads per sample. Raw metagenomic sequencing reads were trimmed and quality filtered with fastq‐mcf software supplied by Illumina Inc (minimum mean quality score, 20; window size, 5 bp; and minimum length, 100 bp). Following quality filtering, an average of 18,662,746 ± 5,671,267 quality‐filtered microbial reads per sample were retained (Table S8).

### *Lactobacillus crispatus* strain‐level profiling of the bioreactor‐derived cultures

To disentangle the different *L. crispatus* strains in the co‐culture aliquots collected at different time points (10, 24, and 48 h) during bioreactor growth, the filtered metagenomic reads obtained from each shotgun sequencing effort were mapped against specific distinctive regions of every *L. crispatus* genome using the software BBMap (https://sourceforge.net/projects/bbmap/) with 100% homology (perfect mode = t flag). Notably, to reduced multi‐mapped metagenomic reads, we ensured that the selected *L. crispatus* genomes returned pairwise ANI values <98%, as advised in a previous qualified study (Olm, 2021) (https://drep.readthedocs.io/en/latest/choosing_parameters.html). In detail, to identify suitable discriminative genes, the whole set of genes unique to each *L. crispatus* strain detected in the PGAP analysis were mapped against the combined genomes of all strains using the Bowtie2 ‐‐very‐sensitive mode (Langmead & Salzberg, 2012). Genes that did not return any hits other than those corresponding to the genome to which they belong were retained as candidate strain‐specific marker genes. This selection was then manually inspected to exclude genes corresponding to transposases, phage genes and genes located alongside the contig ends. This procedure identified a set of roughly ten unique marker genes for each *L. crispatus* strain that were used in downstream analyses on the bioreactor‐derived metagenomic reads (Table S7). A proxy measure of each strain abundance was calculated by normalising the mapped read count on the corresponding marker gene length and library size using the RPKM mathematical formula [(10^9^ * Number of mapped reads to a gene)/(Total mapped reads * gene length in base‐pairs]).

Moreover, the set of genes associated uniquely with each of the four co‐cultivated strains was functionally investigated to discover potential accessory protein‐encoding sequences conferring peculiar growth abilities in the cultivation medium. To this scope, we employed the MetaCyc database (https://metacyc.org/), which allowed us to assign a detailed functional annotation to each scrutinized gene. In addition, the Transporter Classification Database (TCDB) was exploited to characterise transport systems and identify their possible substrates (https://tcdb.org/).

### Statistical analysis

The software SPSS version 25 and OriginPro version 2023 (www.ibm.com/software/it/analytics/spss/) (https://www.originlab.com/) were used for statistical data analyses and graphing. One‐way ANOVA with Bonferroni correction was used to determine the statistical significance of differences in the OD_600_ measures (growth assay) and normalised read counts (bioreactor‐based co‐cultivation experiment). AlphaFold (Jumper et al., 2021) and PyMOL software (https://pymol.org/2/) were used to observe SNPs within the predicted 3D protein structure of the pullulanase type II gene derived from the *L. crispatus* LB97.

## RESULTS AND DISCUSSION

### Identification of representative *Lactobacillus crispatus* genomes

To investigate the genomic differences between human *L. crispatus* strains, an extensive comparative genome analysis was performed on *L. crispatus* genomes recovered from human specimens of healthy donors, including faecal, vaginal, saliva and urine samples (Table S1). Specifically, seven *L. crispatus* strains were obtained from international bacteria culture collection (Table 1), and their genomes were sequenced along with those of 34 strains isolated from the human vaginal tract in the context of a previous study (Table 1). Additionally, with the aim of expanding the overview of the genetic variability of this taxon, 200 genome sequences (complete and draft) of *L. crispatus* strains isolated from human biological samples were selected from public repositories (Table S1). Following dereplication aimed at removing the genomic redundancy by grouping essentially identical genomes (using dRep tool, version 2.2.0, with average nucleotide identity >99%, [https://drep.readthedocs.io/en/latest/choosing_parameters.html]), 22 *L. crispatus* chromosomes with average completeness of 98.97% ± 0.14% were retained as representatives of the sequence variation observed in our genome repertoire and therefore used for comparative analysis (Table S2).

**TABLE 1:** Genome features of the 22 representative *Lactobacillus crispatus* genomes.

Assembly ID	Strain name	Genome size Mbp	CDS number	Genome completeness (%)	Isolation source
GCF_000162255.1	125‐2‐CHN	2.30525	2032	99.03	Human vagina
GCF_000162315.1	MV‐3A‐US	2.43708	2252	98.38	Human vagina
GCF_002861805.1	UMB0824	2.17405	2061	99.03	Human urine
GCF_002861815.1	UMB0085	2.17506	2081	99.03	Human urine
GCF_009857395.1	Indica2	2.20949	2028	99.03	Human vagina
GCF_007713895.1	NCK1350	2.04734	1932	99.03	Human stool
GCF_013456995.1	B4	2.03959	1902	99.03	Human stool
GCF_000160515.1	JV‐V01	2.2172	1992	98.03	Human vagina
GCF_014654865.1	BC5	2.06419	1901	99.03	Human vagina
GCF_015669875.1	D31t1	2.2782	2120	99.03	Human stool
GCF_018987235.1	ATCC 33820	2.23909	2020	99.03	Human saliva
GCF_020042005.1	Lc1700	2.81896	2632	98.86	Human vagina
GCF_021278925.1	lc83	2.30843	2112	98.9	Human vagina
GCF_025194085.1	CIRM‐BIA 2111	2.00737	1865	99.03	Human stool
GCF_025194045.1	CIRM‐BIA 2233	2.24513	2127	99.03	Human vagina
This study	LMG11440	2.032412	2087	98.86	Human vagina
This study	LMG12005	2.019682	2014	98.94	Human vagina
This study	LMG18189	2.094399	2079	99.03	Human saliva
This study	LMG11415	2.030901	2004	99.03	Human saliva
This study	LMG18200	2.208098	2182	99.03	Human stool
This study	LB93	2.202822	2340	99.03	Human vagina
This study	LB97	2.263389	2422	99.03	Human vagina

The general features of the 22 representative *L. crispatus* genomes are reported in Table 1 and include an average of 2105 ± 179 predicted Coding Sequences (CDSs) per chromosome (ranging from 2632 to 1865), with an average genome length of 2.20 ± 0.18 Mbp.

### Intra‐species genetic variability within the *Lactobacillus* genus

**FIGURE 1:**
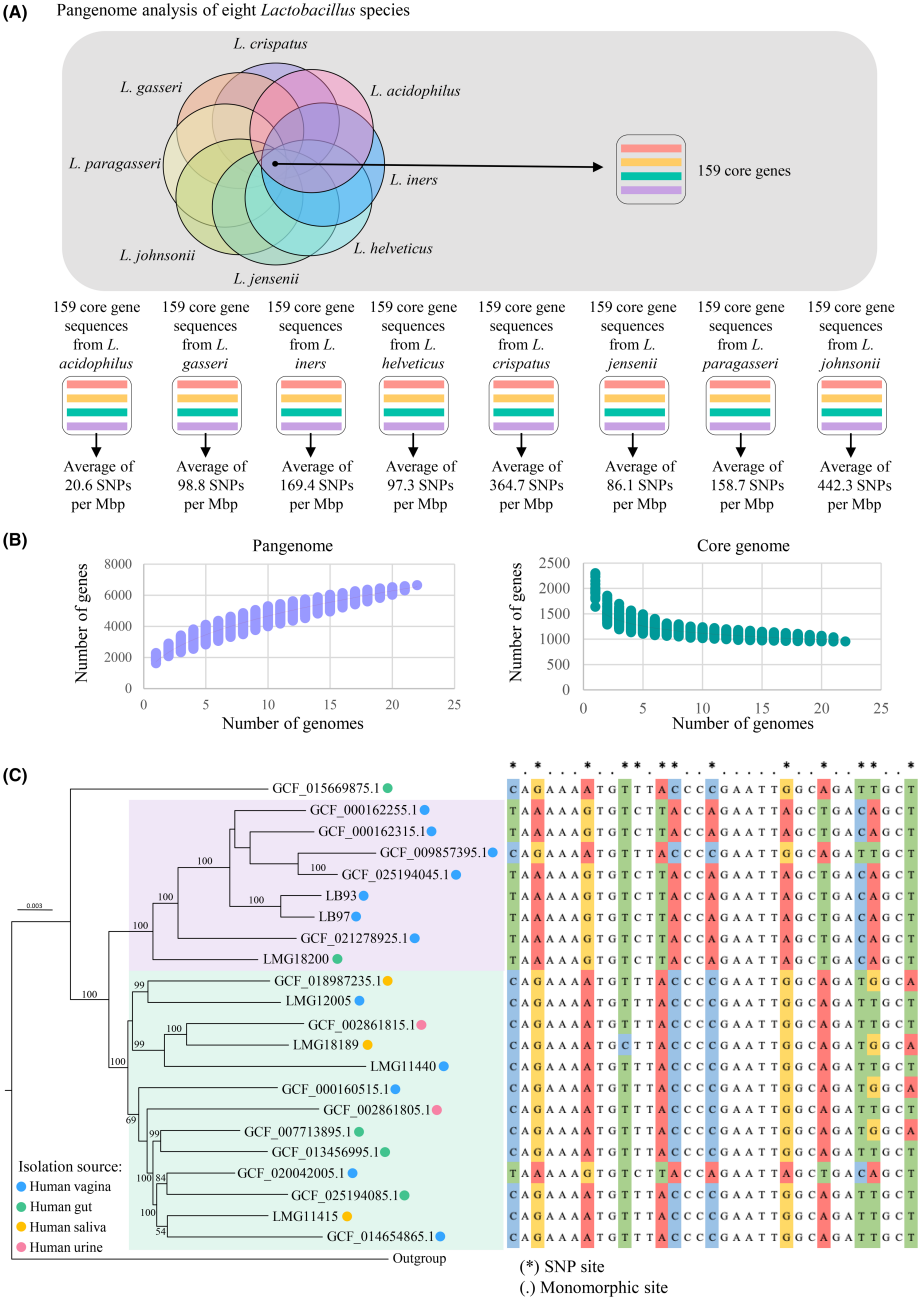
Comparative analysis between different *Lactobacillus* species and between 22 non‐identical *L. crispatus* strains. In panel (A), Venn diagram shows the eight *Lactobacillus* species sharing the 159 genes used to measure the magnitude of intra‐species genetic diversity. Below, the species‐specific number of SNPs identified within the common protein‐encoding genes is reported for each of the considered *Lactobacillus* species. Panel (B) depicts the *L. crispatus* pan‐ and core‐genome size. The number of discovered genes (vertical axe) is plotted as a function of the number of sequentially added genomes (horizontal axe). Panel (C) shows the phylogenomic tree based on the concatenated 959 core genes shared among the 22 non‐identical *L. crispatus* genomes. The tree was constructed by the neighbour‐joining method. Bootstrap percentages based on 1000 replicates above 50 are shown at node points. For each strain, the isolation source is highlighted with a coloured circle. On the right, an aligned portion of the *L. crispatus* core genome exemplifies the relationships between phylogenomic clusters and SNP patterns. In the top row, nucleotide positions showing variants are highlighted with an asterisk, while a dot highlights non‐variant sites.

To investigate the level of genomic diversity among *L. crispatus* strains compared with other species of the *Lactobacillus* genus, we selected publicly accessible chromosomes belonging to seven different *Lactobacillus* species known to inhabit various human body sites. Notably, for a robust comparison with the dereplicated 22 representative *L. crispatus* genomes, we focused on *Lactobacillus* species for which at least 20 independent conspecific genomes with ANI values between 95% and 98% were retained after accounting for genome completeness >95% (Table S3). Accordingly, 499 *Lactobacillus* chromosomes were collected and combined with the 22 representative genomes of *L. crispatus* for pangenome analysis, which led to the identification of 159 core genes, defined as the set of gene families (clusters of orthologous groups [COGs]) shared by each *Lactobacillus* chromosome tested (Figure 1A).

Exploiting this set of 159 core genes, the level of variability at the single nucleotide level was evaluated individually within each *Lactobacillus* species. In detail, sequences homologous to the 159 core genes (corresponding to an average of 46,760 ± 987 nucleotides) were recovered from each collected genome and compared between strains belonging to the same *Lactobacillus* species, eventually recording Single Nucleotide Polymorphisms (SNPs) at each nucleotide position.

Considering the nucleotide variations with an occurrence rate above 20% to exclude sequencing errors, the eight inspected *Lactobacillus* lineages exhibited a total number of intra‐species SNPs per Mbp ranging from 20.6 to 442.3, with *L. johnsonii* and *L. acidophilus* showing the lowest and the highest number of SNPs, respectively (Figure 1A). Thus, these data highlighted how, on average, the various species of the *Lactobacillus* genus display different levels of intra‐species genetic diversity, which has the potential to translate into intra‐species phenotypic variability.

Remarkably, the *L. crispatus* core gene set returned an average of 364.7 SNPs per Mbp, emerging among the species with the higher genetic variation, even compared with other notorious *Lactobacillus* species inhabiting the human vaginal tract, such as *L. gasseri*, *L. iners* and *L. jensenii* (Figure 1A). Specifically, among *L. crispatus* members, most SNPs were found in gene sequences coding for transmembrane transport mechanisms (26%), followed by genes predicted to be involved in the biosynthesis of extracellular protein components (14.5%) and carbohydrate metabolism (10.4%). In contrast, within more niche‐specialised *Lactobacillus* species, for example *L. gasseri*, the genes with higher number of SNPs were predicted to be involved in DNA‐related processes (21.4%) and protein‐protein interaction (12%).

### Pan‐ and core‐genome analysis of the *L. crispatus* species

Chromosome sequences of the 22 non‐identical *L. crispatus* strains were submitted to gene re‐annotation and subsequently analysed from a pangenome perspective, providing information on the ubiquitous genetic backbone of conspecific chromosomes and the intra‐species genetic diversity (Tettelin et al., 2005). In total, the pangenome of *L. crispatus* includes 6512 COGs, whose accumulation curve, depicting the expansion of the pangenome as a function of the number of genomes included, is still far from being saturated. Thus, indicating that *L. crispatus* species is characterised by an open pangenome where the total gene pool obtainable for this species has not yet been fully disclosed (Figure 1B). Moreover, we determined the current *L. crispatus* core genome to be comprising 959 COGs that were conserved across all the 22 analysed strains (15% of the pangenome) while an average of 157.3 ± 54.3 genes per genome were associated with only one strain.

Based on the core gene sequences obtained from the 22 non‐redundant *L. crispatus* genomes, a phylogenetic tree was constructed to evaluate the evolution of the species (Figure 1C). According to the clustering relationship, the 22 strains assessed were divided into two main clusters, one of which was intriguingly composed only of *L. crispatus* strains isolated from the female reproductive tract (Figure 1C, violet shadows). Moreover, this phylogenetic tree also displayed a second phylogenetic cluster of strains isolated from different human body districts, encompassing vagina, gut, saliva and urine (Figure 1C, green shadow). Notably, this mixed group may include a few strains that can survive/colonise in closely related niches, like different human body districts.

To investigate the intra‐species genomic variability of *L. crispatus* taxon, we measured the genetic diversity at the single‐nucleotide level by comparing the whole genome sequences (wgSNPs) and the corresponding core genome (cgSNPs). Specifically, from the core genome‐based phylogenomic tree (Figure 1), we selected the *L. crispatus* strain placed at the deepest split, that is the most divergent chromosome (RefSeq genome assembly GCF_015669875), which was used as a reference sequence to compute pairwise alignments and SNPs extraction. Overall, the 22 *L. crispatus* strains showed an average of 28,811 wgSNPs (representing about 2% of the genome sequence), most of which (about 90%) resided within CDSs.

For cgSNPs evaluation, each of the 22 homologous nucleotide sequences obtained by concatenating the 959 COGs shared among all the non‐identical *L. crispatus* strains (corresponding to an average of 904,903 bases) was examined for sequence variations against the concatenated (core) gene set of the reference *L. crispatus* assembly. This procedure resulted in the identification of an average of 15,007 cgSNPs (representing a variation rate of 1 for every 56 nucleotides) that was lowered when compared with previous analyses of polymorphic sites within clinically relevant microorganisms such as *Pseudomonas aeruginosa* and *Escherichia coli* (showing 159,609 SNPs within the concatenated core genes of 3,629,979 bp and 266,969 SNPs within a core genome of 2,159,296 bp, respectively) (Muthukumarasamy et al., 2020; Shakya et al., 2020). Albeit the core genome resulted rather conserved within these 22 *L. crispatus* strains, it might be worth mentioning that the observed micro‐diversity lies within DNA sequences that code for proteins. Therefore, it could have evolutionary importance contributing to intra‐species diversity by affecting protein domains. Indeed, the nucleotide sequence variation among the *L. crispatus* core genome was not randomly distributed, but phylogenetically co‐clustered strains showed common patterns of SNP profiles (Figure 1C), thus indicating that the observed SNPs are representing evolutionary trajectories and not mere random mutations or sequencing errors.

### Exploration of the micro‐diversity in the ubiquitous features of *L. crispatus* genome and identification of fast evolving genes

With the aim of defining whether and which categories of genes are more concerned by a rapid sequence evolution, we calculated the level of polymorphisms for each protein‐coding gene constituting the *L. crispatus* core genome. Like what has been performed above, the homologous gene sequences from the *L. crispatus* GCF_015669875 were used as reference in pairwise comparisons of each individual core gene. Accordingly, the number of SNPs resulting from the average of all alignment pairs ranged from zero to 347.45 ± 150.30 per gene (Table S4).

**FIGURE 2:**
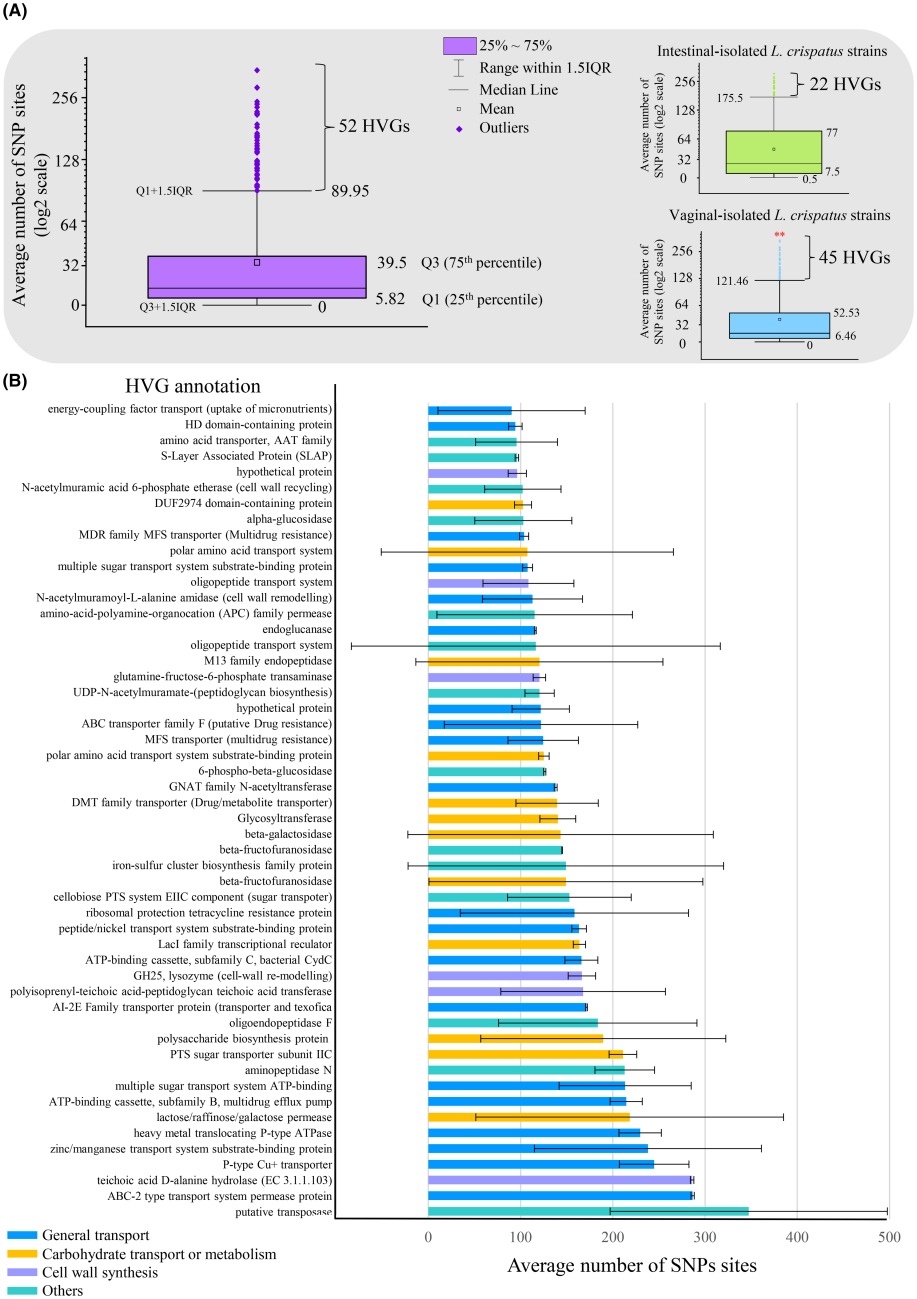
Identification of the 52 HVGs. In panel (A), Box‐Whisker plot was used to represent the gene distribution based on the number of SNP sites obtained by comparing the nucleotide sequence of every 959 protein‐coding genes shared among all the 22 *Lactobacillus crispatus* chromosomes. For each gene, the number of SNP sites was expressed as the average of all the pairwise comparisons against the reference sequence (homologous gene sequence of *L. crispatus* GCF_015669875). The Q3 + 1.5IQR was used as a cut‐off to select the 52 HVGs. Panel (B) reports the functional annotation and the number of SNP sites of each HVG.

Among the genes with the lower average number of SNP sites (lower than 5.8, corresponding to the data below the 25th percentile, Figure 2A), we identified protein‐coding sequences involved in putative housekeeping functions, including ribosome assembly and function and central glycolysis regulation, as well as DNA replication and cell division (Table S4).

In contrast, by considering the data above the third quartile (Q3 + 1.5·Inter‐Quartile Range, Figure 2A), we identified 52 genes with the highest average number of SNP sites (ranging from 347.45 ± 150.30 to 90.25 ± 28.76), that is the most highly variable genes (HVGs), which therefore represented the set of genes that have been likely under the strongest selection pressure (Figure 2B, Table S4). Interestingly, among the HVGs, we identified mainly genes involved in the biosynthesis and rearrangement of cell wall components, such as lipoteichoic acids and peptidoglycan, as well as transmembrane transport of a variety of substrates, including carbohydrates and micronutrients (Figure 2B, Table S4). The presence of such micro‐diversity in proteins directly mediating interactions with the environment likely reflects adaptive mechanisms to the changing biotic and abiotic components, thereby leading to possible different competitive abilities and (sub‐) niche specialisation. Indeed, the intra‐species heterogeneity observed in the *L. crispatus* core genes emerged less marked when the genomes of 22 *L. crispatus* strains were compared based on their ecological niche, thus showing greater gene sequence homogeneity among genomes sharing the same environment (Figure 2A). However, vaginal‐derived *L. crispatus* strains showed a significantly higher number of HVGs than those isolated from the human gut (Mann–Whitney test, *p*‐value = 0.007), indicating that the vaginal environment exerts crucial ecological forces driving the *L. crispatus* genome evolution.

**FIGURE 3:**
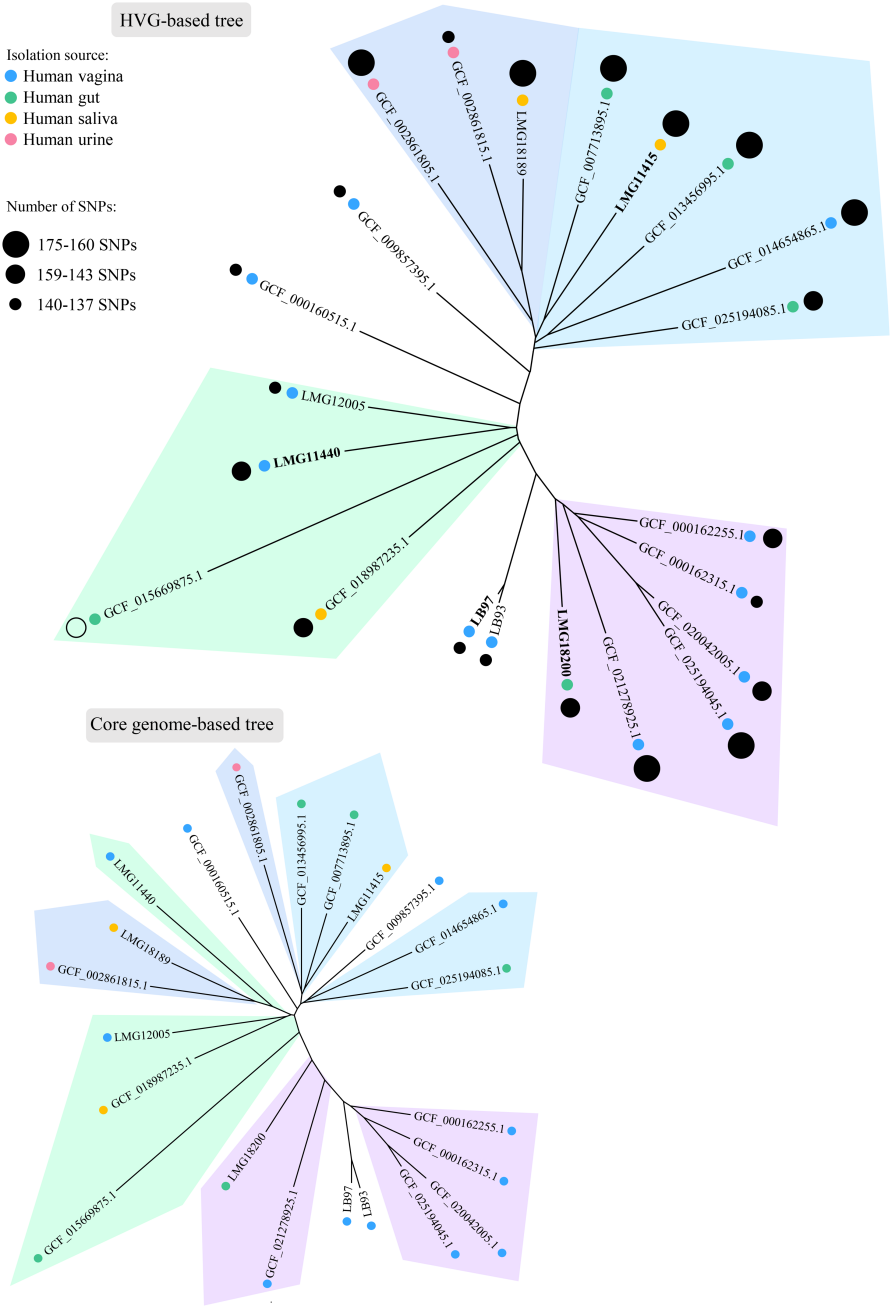
Phylogenetic analysis based on the 52 HVG sequences. Proteomic tree based on concatenating the 52 protein encoding core genes identified as highly variable across the 22 non‐identical *Lactobacillus crispatus* genomes. Phylogenetic groups are highlighted in different colours. For comparison, the phylogenomic tree resulting from the whole core genome (presented in Figure 1) is visualised using the redial layout. For each strain, the coloured circle represents the isolation source, while the diameter of the black circle is proportional to the number of SNPs identified within the core genome using the GCF_015669875.1 genome sequence as reference.

A new phylogenomic tree, representing the evolutionary outcomes determined by mutational hotspots within the *L. crispatus* species, was generated employing the nucleotide sequences of the 52 HVGs (Figure 3). Specifically, this tree was composed of three main clusters, where not all the strains maintained the same distribution compared to the original phylogenomic tree based on the whole core genome (Figure 3). Indeed, the HVG‐based tree better distinguished among strains from closely related niches, highlighting that the evolution of the HVGs does not strictly follow the overall strain speciation, probably reflecting a relatively recent adaptation to specific environmental stimuli, such as multiple human body site colonisation or inter‐strain niche competition. Accordingly, based on the picture emerging from the HVG‐derived phylogenetic distribution, we selected four highly divergent *L. crispatus* strains, that is LB97, LMG11440, LMG18200 and LMG11415, which were used for in vitro phenotypical assays aimed at investigating the link between evolutionary trajectories, grow performances and competitive abilities.

### In vitro evaluation of *L. crispatus* growth performances on selected carbohydrate sources in an in vitro human vaginal model

**FIGURE 4:**
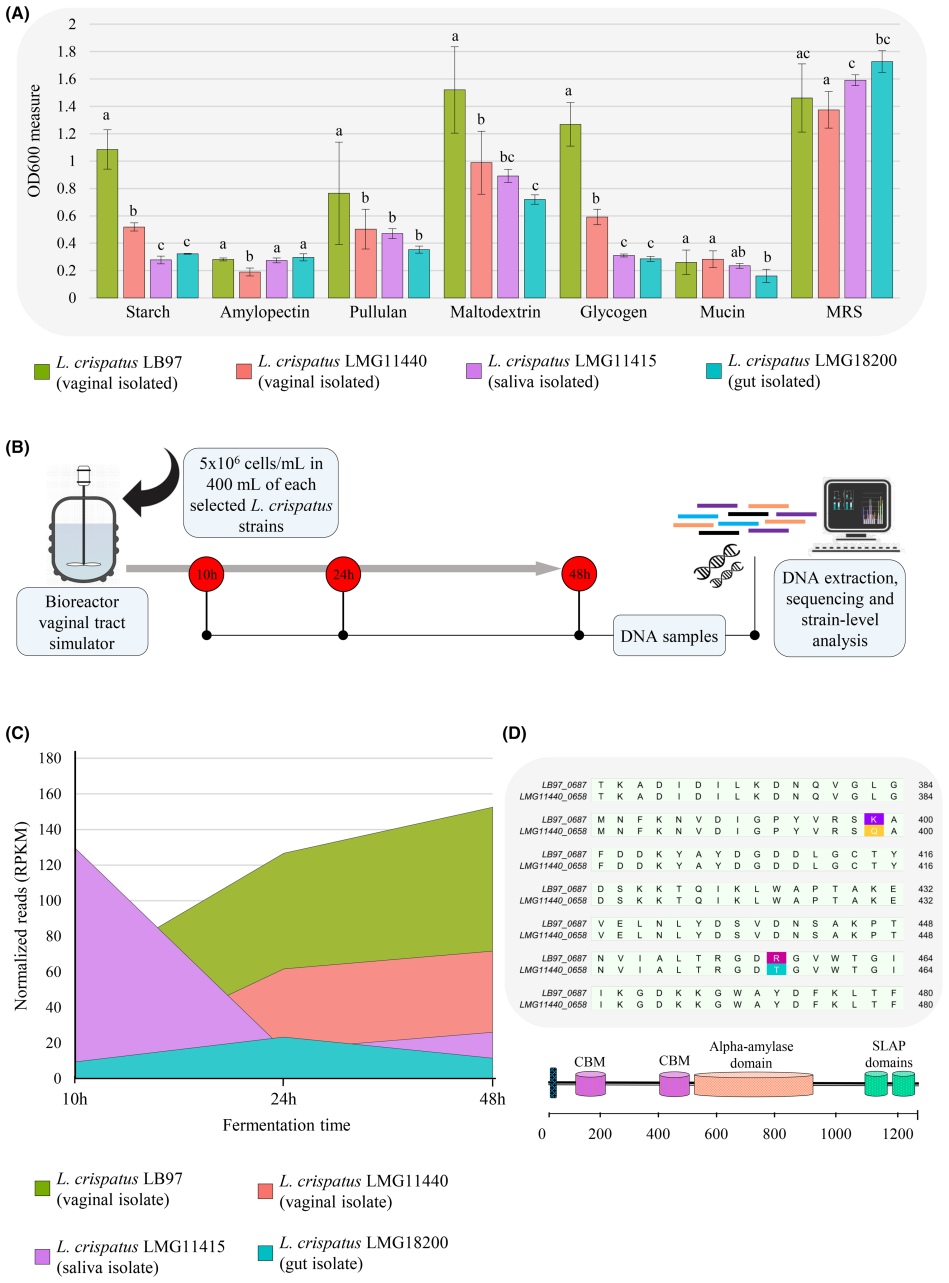
Differential growth and competitive abilities between *Lactobacillus crispatus* strains. Panel (A) shows the optical density (OD) registered after 48 h of anaerobic growth in different nutritive substrates. Panel (B) illustrates the design of the bioreactor‐based experiment simulating the vaginal environment. In panel (C), the bar chart reports the quantification of the metagenomic reads (using average RPKM measures) mapping marker genes unique to each *L. crispatus* strain throughout the 48 h of growth in the bioreactor. The standard deviations are plotted as error bars. Different lowercase letters indicate significant differences at *p*‐value < 0.05 according to the Bonferroni test. In panel (D), alignment of partial amino acid sequences corresponding to the type II pullulanase genes of *L. crispatus* LB97 and LMG11440 strains genes highlights two amino acid substitutions Gln (Q) to Lys (K) and Arg (R) to Thr (T).

To investigate how the high level of genetic heterogeneity identified within *L. crispatus* could influence the respective growth abilities, the four selected *L. crispatus* strains (LB97, LMG11440, LMG18200 and LMG11415, whose genome diversity corresponded to ANI pairwise values <98%, Table S2) were cultivated on six different carbohydrate sources including glycogen, which is the primary bacterial nutritional source in the vaginal lumen (Tester & Al‐Ghazzewi, 2018; Zhang et al., 2022), along with other glycogen‐like α‐glucans, which may also represent a substrate for the bacterial enzymatic arsenal involved in carbohydrates breakdown of the vaginal environment (Figure 4A, Table S5). The optical density (OD) was registered after 48 h of anaerobic cultivation, and the growth on MRS was used as control condition (Table S5). Upon one‐way ANOVA test with Bonferroni correction (cut‐off *p*‐value < 0.05), the comparative growth assay showed widespread statistically significant differences across the four *L. crispatus* strains. In particular, *L. crispatus* LB97, isolated from the human vagina, showed greater growth performances on most of the carbohydrates tested, including glycogen (final OD >1.2; all Bonferroni‐corrected *p*‐values < 0.05), thus demonstrating a metabolic specialisation consistent with its isolation niche. Conversely, the gut‐derived *L. crispatus* LMG18200 exhibited the lowest growth when glycogen, starch and pullulan were used as the unique carbon source (all final OD measures ~0.3; all Bonferroni‐corrected *p*‐values < 0.05), suggesting the incapability of this strain to metabolise long‐chain α‐glucans (Figure 4A, Tables S5 and S6). Moreover, all strains appeared nearly equally limited in mucin utilisation (all final OD <0.3; Bonferroni‐corrected *p*‐values > 0.05; Figure 4A, Tables S5 and S6).

Furthermore, to determine the reciprocal competitive ability of the different *L. crispatus* strains, the growth performances of the four selected strains were evaluated through a co‐cultivation experiment involving a bioreactor model simulating the nutritional and chemical–physical conditions of the vaginal environment (Pan et al., 2020) (Figure 4B). The proliferation trend of each strain was followed for 48 h by mapping the sequenced metagenomic reads at multiple time points against a set of strain‐specific marker genes (Tables S7 and S8). In accordance with what was observed in the carbohydrate grow assay, the vaginal isolate LB97 showed a notable proliferation ability, over dominating the four‐strain *L. crispatus* community at every co‐cultivation time‐point (Table S8, all Bonferroni‐corrected *p*‐values < 0.05; Figure 4C).

In contrast, the strains LMG11415 (isolated from the human saliva) and LMG18200 (isolated from the human intestine) were clearly overwhelmed (Figure 4C). Consistently, close examination of the genomes of these four *L. crispatus* strains revealed that these latter were lacking in any glycogen‐degrading encoding gene while the proliferating vaginal‐derived LB97 and LMG11440 strains carried a type II pullulanase acting on both α‐1,6‐ and α‐1,4‐glycosidic bonds, which therefore achieves complete glycogen degradation, as reported in recent studies (van der Veer et al., 2019; Zhang et al., 2022). Nevertheless, LB97 and LMG11440 substantially differ in their proliferation capabilities (Table S8, Bonferroni‐corrected *p*‐values < 0.05; Figure 4C).

Accordingly, the metabolic potential of the gene sequences identified in the comparative genome analysis as associated uniquely with the *L. crispatus* LB97 was investigated using the MetaCyc database (Caspi et al., 2018). The results revealed that the predicted proteome of this strain is characterised by the presence of protein‐encoding genes involved in the uptake and metabolism of galactitol and polyamines, as well as a locus‐encoding proteins dedicated to the ascorbate degradation, which can contribute to the maintenance of the host's vaginal health (Linares et al., 2011) (Table S9). Moreover, the unique gene repertoire of the LB97 strain also contained a gene encoding for a mucin‐binding protein (MucBP domain), thus corroborating for this strain the hypothesis of a host mucin role as adhesion site rather than a carbon source, as previously reported in the literature (Muthukumarasamy et al., 2020; Shakya et al., 2020; Tester & Al‐Ghazzewi, 2018) and confirmed by the growth assay described above (Figure 4A). However, our functional investigation did not detect genes possibly involved in the metabolism of the nutritional sources constituting the used glycogen‐based culture medium (Table S9).

Interestingly, when SNPs were calculated between these latter two *L. crispatus* genome sequences, a total of 27,906 nucleotide positions showed differences of which 8238 (29% of the total SNPs) corresponded to amino acid replacements, thus resulting in strain‐specific intragenic variants, which can contribute to generating phenotypic differences (Table S10). Moreover, alignment of their type II pullulanase gene revealed four variations at single nucleotide level (Table S11, Figure S1), two of which resulted in amino acid substitutions (Figures 4D and S1). In more detail, these non‐synonymous SNPs lie within the protein Carbohydrate Binding Module (CBM) (Figure 4D), with possible repercussions on the efficiency of the protein binding to its substrate, as also evidenced by the 3D protein structure prediction (Figure S2).

Overall, the finding of isolate‐specific intragenic SNPs, and particularly those within the pullulanase‐encoding gene, possibly explains the growth and competitiveness differences observed between the vaginal‐isolated *L. crispatus* LB97 and LMG11440 strains cultivated on the simulated vaginal medium.

## CONCLUSION

In this study, a comparative genome analysis involving 41 newly decoded human *L. crispatus* genomes coupled with 200 publicly available genome sequences from this species allowed us to deeply investigate the *L. crispatus* core gene evolution by connecting data from single nucleotide variations, phylogenomic reconstructions and in vitro experiments.

Compared with other *Lactobacillus* species, including those inhabiting the human vaginal tract, that is *L. iners, L. gasseri* and *L. jensenii*, a higher level of sequence variation at the single nucleotide level was observed within the gene pool shared among the inspected *L. crispatus* strains, thus highlighting a within‐species diversity driven by conserved genes evolution.

Interestingly, the genetic heterogeneity observed within the *L. crispatus* species appears to be reflected at the phenotypic level. In fact, when different *L. crispatus* strains were co‐cultivated in a bioreactor‐based model simulating the vaginal environment, substantial differences were noted in the colonisation and competition efficiency. Although members of the *L. crispatus* species were previously thought to utilise the glycogen hydrolysis products generated in the vaginal environment by the human α‐amylase (Mirmonsef et al., 2014; Tester & Al‐Ghazzewi, 2018), recent evidences showed that members of this taxon produce the enzyme to independently degrade glycogen, annotated as a type II pullulanase (van der Veer et al., 2019; Zhang et al., 2022). In this context, while the absence of this gene was noted for those *L. crispatus* strains unable to stably proliferate on glycogen under in vitro conditions, we identified two amino acid substitutions within the type II pullulanase carbohydrate‐binding module arising from non‐synonymous SNPs, which could explain the different proliferation and dominance abilities observed in vitro for the *L. crispatus* strains investigated in this study.

Remarkably, while the strain‐specific accessory genetic content has been historically pointed out as one of the main sources of variability resulting from intra‐species evolution, data collected in the framework of this study revealed that the evolution of the core genome could contribute to generate marked strain‐specific phenotypic traits. Thus, understanding this evolutionary driving force could be relevant for unravelling strain‐specific capabilities to successfully dominate the female reproductive tract and, ultimately, selecting suitable *L. crispatus* strains that could be applied for novel bacterial therapy strategies.

## AUTHOR CONTRIBUTIONS

**Chiara Tarracchini:** Data curation (lead); formal analysis (lead); investigation (lead); methodology (equal); software (equal); writing – original draft (lead). **Chiara Argentini:** Data curation (equal); investigation (equal); validation (equal). **Giulia Alessandri:** Data curation (supporting); methodology (supporting). **Gabriele Andrea Lugli:** Data curation (supporting); software (equal). **Leonardo Mancabelli:** Data curation (supporting); supervision (supporting). **Federico Fontana:** Data curation (supporting); software (supporting). **Rosaria Anzalone:** Investigation (supporting). **Alice Viappiani:** Investigation (supporting). **Francesca Turroni:** Conceptualization (equal); project administration (supporting); supervision (supporting); writing – review and editing (supporting). **Marco Ventura:** Conceptualization (lead); project administration (lead); writing – review and editing (lead). **Christian Milani:** Conceptualization (supporting); data curation (supporting); methodology (supporting); project administration (supporting); supervision (lead); writing – review and editing (equal).

## CONFLICT OF INTEREST STATEMENT

The authors declare no conflict of interest.

## Supporting information


Figure S1.

Figure S2.

Table S1.
Table S2.
Table S3.
Table S4.
Table S5.
Table S6.
Table S7.
Table S8.
Table S9.
Table S10.
Table S11.

## Data Availability

Genome sequences of the 41 newly sequenced *L. crispatus* were deposited in NCBI‐SRA (Short Read Archive) repository with accession number PRJNA947599.
