# Supplementary figures and images for: The core genome evolution of Lactobacillus crispatus as a driving force for niche competition in the human vaginal tract
Source: Microb Biotechnol. 2023 Jul 25;16(9):1774–89. doi: 10.1111/1751-7915.14305 (PMC10443340; doi:10.1111/1751-7915.14305)

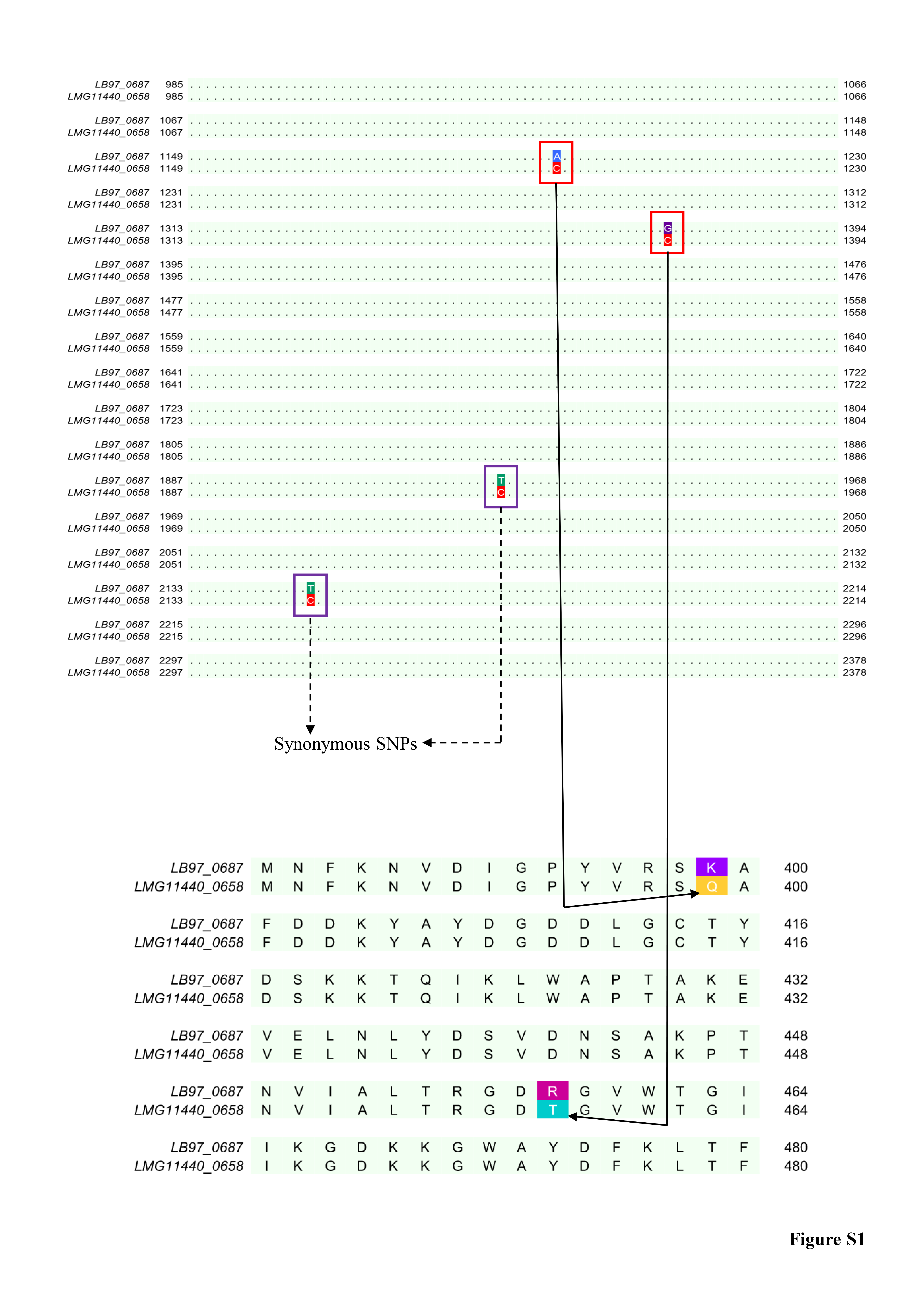

Supplement: Supplementary file 1 — Figure S1. [file MBT2-16-1774-s002.png]

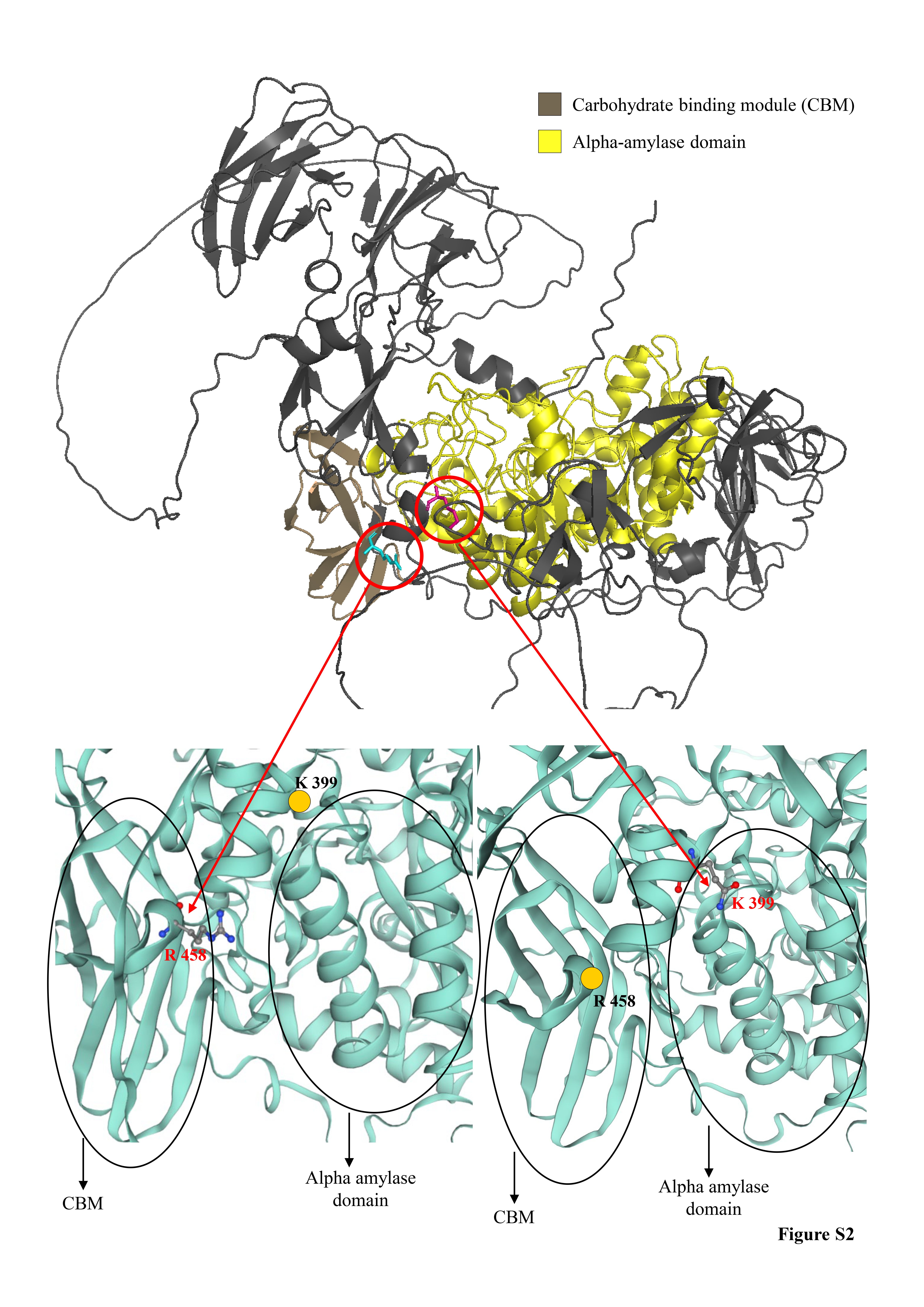

Supplement: Supplementary file 2 — Figure S2. [file MBT2-16-1774-s001.png]
